# Incidence, prevalence and mortality of chronic liver diseases in Sweden between 2005 and 2019

**DOI:** 10.1007/s10654-023-01028-x

**Published:** 2023-07-25

**Authors:** Patrik Nasr, Erik von Seth, Raphaela Mayerhofer, Nelson Ndegwa, Jonas F. Ludvigsson, Hannes Hagström

**Affiliations:** 1 Department of Medicine, Huddinge, Karolinska Institutet, Stockholm, Sweden; 2 Department of Gastroenterology and Hepatology, Department of Health, Medicine and Caring Sciences, Linköping University, Linköping, Sweden; 3 Division of Hepatology, Department of Upper GI Diseases, Karolinska University Hospital, 141 86 Stockholm, Sweden; 4 Department of Medical Epidemiology and Biostatistics, Karolinska Institutet, Stockholm, Sweden; 5 Celiac Disease Center, Department of Medicine, Columbia University Medical Center, New York, NY USA; 6 Department of Pediatrics, Örebro University Hospital, Örebro, Sweden

**Keywords:** Cirrhosis, Hepatocellular carcinoma, Epidemiology

## Abstract

**Background:**

Updated data on the incidence, prevalence, and regional differences of chronic liver disease are missing from many countries. In this study, we aimed to describe time trends, incidence, prevalence, and mortality of a wide range of chronic liver diseases in Sweden.

**Methods:**

In this register-based, nationwide observational study, patients with a register-based diagnosis of chronic liver disease, during 2005–2019, were retrieved from the Swedish National Board of Health and Welfare. Annual age-standardized incidence and mortality rates, and prevalence per 100,000 inhabitants was calculated and stratified on age, sex, and geographical region.

**Results:**

The incidence of alcohol-related cirrhosis increased by 47% (2.6% annually), reaching an incidence rate of 13.1/100,000 inhabitants. The incidence rate of non-alcoholic fatty liver disease and unspecified liver cirrhosis increased by 217% and 87% (8.0 and 4.3% annually), respectively, reaching an incidence rate of 15.2 and 18.7/100,000 inhabitants, and a prevalence of 24.7 and 44.8/100,000 inhabitants. Furthermore, incidence rates of chronic hepatitis C declined steeply, but liver malignancies have become more common. The most common causes of liver-related mortality were alcohol-related liver disease and unspecified liver disease.

**Conclusion:**

The incidence rates of diagnosed non-alcoholic fatty liver disease, alcohol-related cirrhosis, unspecified liver cirrhosis, and liver malignancies have increased during the last 15 years. Worryingly, mortality in several liver diseases increased, likely reflecting increasing incidences of cirrhosis in spite of a decreasing rate of hepatitis C. Significant disparities exist across sex and geographical regions, which need to be considered when allocating healthcare resources.

**Supplementary Information:**

The online version contains supplementary material available at 10.1007/s10654-023-01028-x.

## Background

Chronic liver disease affects more than 800 million individuals and causes an estimated two million deaths per year worldwide [1]. The most common causes of liver-related mortality are chronic hepatitis B (HBV), chronic hepatitis C (HCV), alcohol-related liver disease (ALD) and non-alcoholic fatty liver disease (NAFLD) [2]. With the availability of curative treatment for HCV and effective vaccines for HBV, the burden of viral hepatitis in high-income countries is decreasing [3]. However, with a six- and twofold increase in the prevalence of obesity [4] and type 2 diabetes mellitus [5] since the 1980s, the hepatic manifestation of the metabolic syndrome, NAFLD, is posing an imminent global health issue [6–9]. The corresponding data in Sweden are slightly attenuated, with an increase in obesity and type 2 diabetes from 5 to 16% and 4.5 to 6.5%, respectively, between 1980 to 2021 [5, 10]. NAFLD progresses slowly and only a small proportion of patients develop liver-related outcomes [11]. However, the total number of individuals with NAFLD is substantially higher than for other chronic liver diseases, leading to many individuals developing cirrhosis. As a result, NAFLD is now the second most common indication for liver transplantation in the US, and among the leading causes of hepatocellular carcinoma (HCC) [7, 12–14].

Also, ALD is increasing both in the US and in the EU [15, 16], with Europe having the highest per capita alcohol consumption in the world [17]. Nevertheless, there are major national differences in Europe, where significant shifts in liver mortality have occurred since the 1970s, which could be attributed to several behavioral, cultural and political changes, such as interventions to reduce alcohol consumption (*e.g.,* fiscal policies, marketing restrictions, and screening initiatives), obesity (*e.g.*, nutritional labeling, food taxes and school- or family-based interventions), and viral hepatitis (*e.g.,* vaccination, access to testing and syringe service programs) [2, 16].

Most studies on chronic liver diseases focus on mortality rather than incidence of non-fatal events. This is mainly attributed to the higher availability of mortality data. Therefore, incidence and prevalence of chronic liver disease, as well as regional (*e.g.,* rural *vs.* urban settings) differences are not well-known. Additionally, studies on the incidence, prevalence, and mortality of rare chronic liver diseases (*e.g.,* autoimmune hepatitis [AIH] and primary biliary cholangitis [PBC]) are rarely reported. Finally, few studies have examined the epidemiological landscape of chronic liver diseases for several diagnoses, mostly instead focusing on single etiologies such as ALD or HCV [18].

In this study, we aimed to describe time trends, incidence, and prevalence of a wide range of chronic liver diseases as well as their role in mortality in Sweden, stratified on categories of age, sex, and geographical locations.

## Methods

### Setting

This register-based, nationwide observational study was conducted in Sweden, with 8,110,944 adults (≥ 18 years) in 2019 (with a total population, including all ages, of 10,327,589 inhabitants in December 2019). All Swedish residents are issued a unique personal identity number (PIN), which permits linkage across several national registries [19]. Universal health care is tax-supported and generally free of charge, which allows for equal access to health care [20].

At health care visits, the responsible physician determines the diagnosis according to International Classification of Disease (ICD) codes for the main and any secondary diagnoses. These ICD codes are regularly transferred to nationwide registers, which are consistently updated. Since 1997, ICD-10 codes have been used in Sweden [21].

The Swedish National Patient Register (NPR) was established on a nationwide level in 1987 and includes all inpatient contacts (hospitalizations). Since 2001, the register also covers all outpatient contacts in specialized care but does not capture primary care. The NPR records all, *i.e.,* both principal and associated diagnoses, made during in- and outpatient contacts. The validity of diagnoses in the NPR is high, with positive predictive values ranging from 85 to 95% [21] for most chronic diseases, 93% for ALD-cirrhosis, and 91% for unspecified cirrhosis [22].

The Swedish Causes of Death Register (CDR) contains cause of death data for all individuals in Sweden [23]. Individuals that have died abroad are also included. It is mandatory for the responsible physician to report the underlying cause of death, and any disease that could have contributed.

The Swedish Cancer Register (SCR) was founded in 1958 and contains data on solid and non-solid tumors. The coverage rate of the register is estimated at approximately 96% [24].

### Study population

All results were based on data obtained from the Swedish National Board of Health and Welfare. Modern ICD-coding of malignancies (*i.e.,* ICD for Oncology version 3 [ICD-O-3]) was introduced in Sweden in 2004–2005. To harmonize the settings and include modern ICD-coding, data was obtained from January 1st, 2005, to December 31st, 2019.

Data was obtained in an aggregated manner, *i.e.,* capturing the total number of individuals with the diagnosis in question. We stratified patients by age groups (18–39, 40–49, 50–59, 60–69, and 70 + years), sex (male and female) and county (n = 21). We identified all adults (≥ 18 years of age) with a first diagnosis of each examined liver disease. The definition of the liver diagnoses was based on the ICD-10 codes presented in Supplementary Table 1. If there were fewer than five individuals per data cell, data was set to missing to protect individual integrity. Patients with a previous ICD-10 code for the diagnosis in question (*i.e.,* between 1997 and 2004) were excluded from incidence calculations. Thus, we only report new cases with each specific liver disease during the study period.

Data on specific etiologies of cirrhosis can in the ICD-10 system only be obtained for three etiologies; HCV (B18.2G or B18.2E), HBV (B18.1G or B18.1E), and ALD (K70.3). Other causes of cirrhosis are assigned the ICD-10 for unspecified cirrhosis (K74.6), hence not allowing for identification of the etiology of cirrhosis in such cases when using aggregated data.

### Statistical analysis

The prevalence, incidence, and mortality rates were all calculated per 100,000 inhabitants. Furthermore, the annual population size was based on the mid-year population per year, calculated as the population at 1^st^ of January and 31^st^ of December, divided by two.

The prevalence of each diagnosis in Swedish adults was calculated by dividing the number of individuals with the diagnosis in question alive on 31^st^ of December 2019, assigned at any time between 2005 and 2019 (as recorded in the NPR or the SCR), with the mid-year adult Swedish population alive in 2019.

The incidence rate of each liver disease was calculated as the number of unique individuals with a first diagnosis of each disease during one calendar year (definitions by ICD codes listed in Supplementary Table 1), divided by the mid-year adult population for that year.

Mortality data was obtained from the CDR and defined as the underlying cause of death. We did not consider contributing causes, since that might lead to individuals being counted twice (for instance, HCC as the main cause of death and ALD-cirrhosis as a contributing cause of death would lead to the same person being counted as two cases of death when using aggregated data).

Annual age- and sex-standardized incidence and mortality rates were calculated per 100,000 inhabitants. Age- and sex-standardized incidence and mortality rates were calculated applying the direct standardization method [25] using the mid-year adult Swedish population as the standard population with age categorized into five groups: 18–39, 40–49, 50–59, 60–69, 70 + . Incidence and mortality rate data is presented as number of deaths per 100,000 inhabitants. In a separate analysis we calculated the mean incidence rate for the full study period stratified on age of diagnosis, and finally separately on county of residence.

The 95% confidence intervals (CI) for directly standardized rates were estimated using a method based on the gamma distribution, a method shown to be suitable when there is a low number of counts [26]. Directly standardized rates were compared by ratios with the female population as the reference in computing rate ratios.

Increase or decrease in rates were visually assessed and if a linear trend was observed, crude percentage increase or decrease was calculated between two timepoints. Crude percentages of increase or decrease in rates between two years were calculated by dividing the rates at the different timepoints. Also, the crude annual percentage of increase or decrease in incidence rate and mortality rate was calculated by $\sqrt[x]{y}$, where *y* is the crude percentage increase or decrease, and *x* is the number of years between the two time points.

The programming language R (version 4.0.5 and 4.2.3) was used to perform the calculations.

## Results

### Incidence and prevalence

**Fig. 1:**
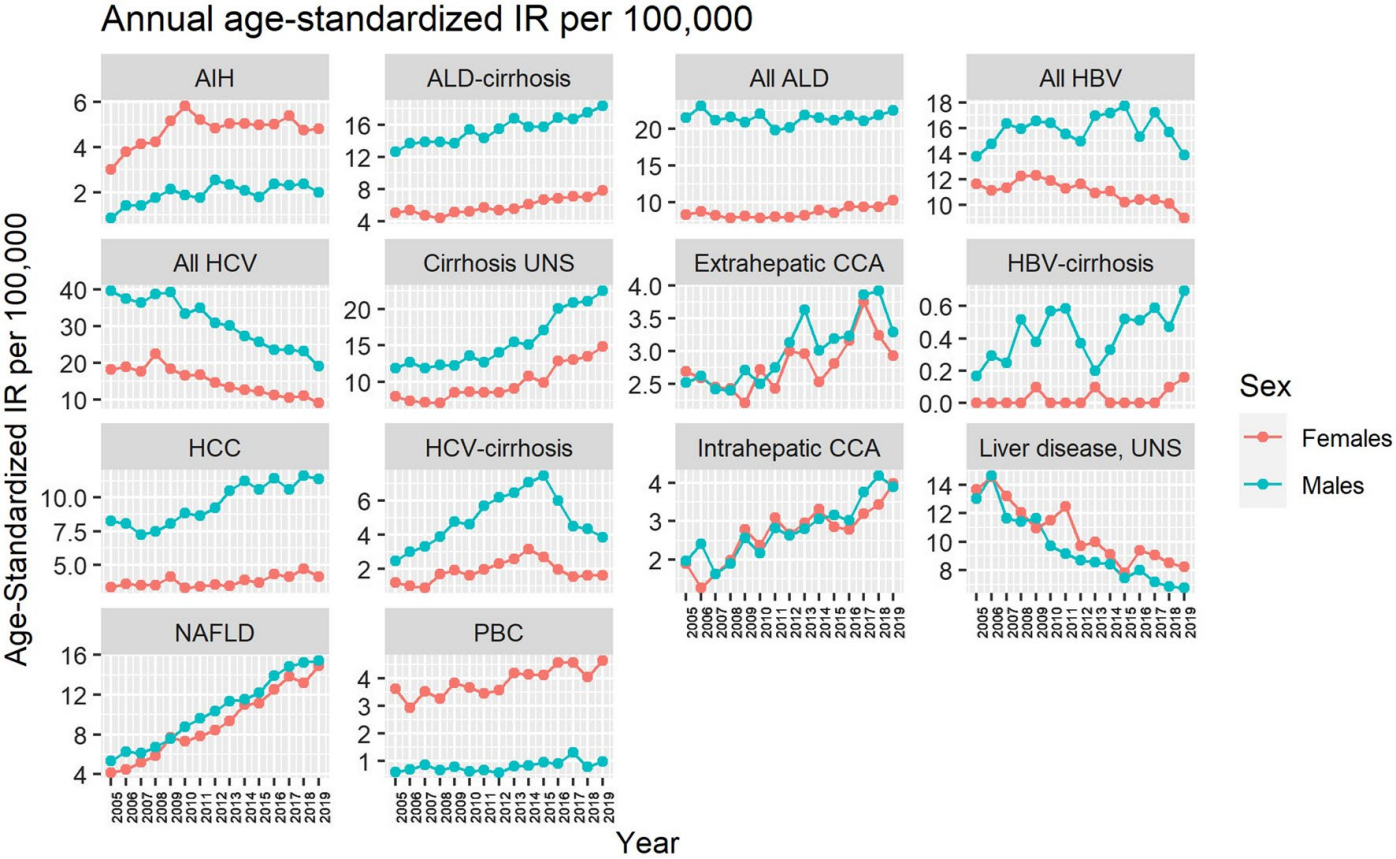
Annual age-standardized incidence rate per 100,000 inhabitants for each diagnosis, stratified on sex, during 2005–2019. Note, different scales are used on the y-axes

**Fig. 2:**
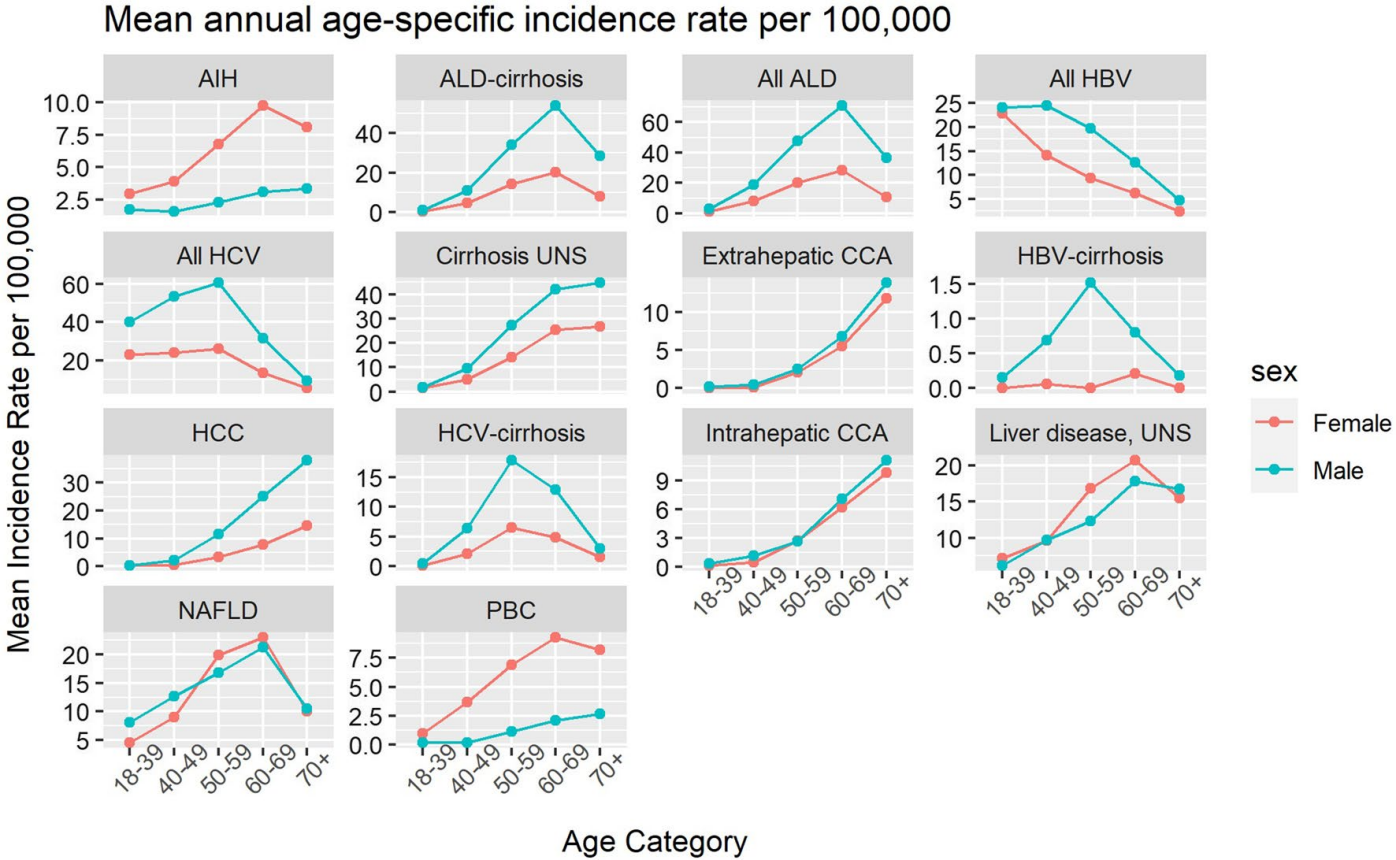
Mean annual age-specific incidence rate per 100,000 inhabitants for each diagnosis, stratified on sex. Note, different scales are used on the y-axes

**Fig. 3:**
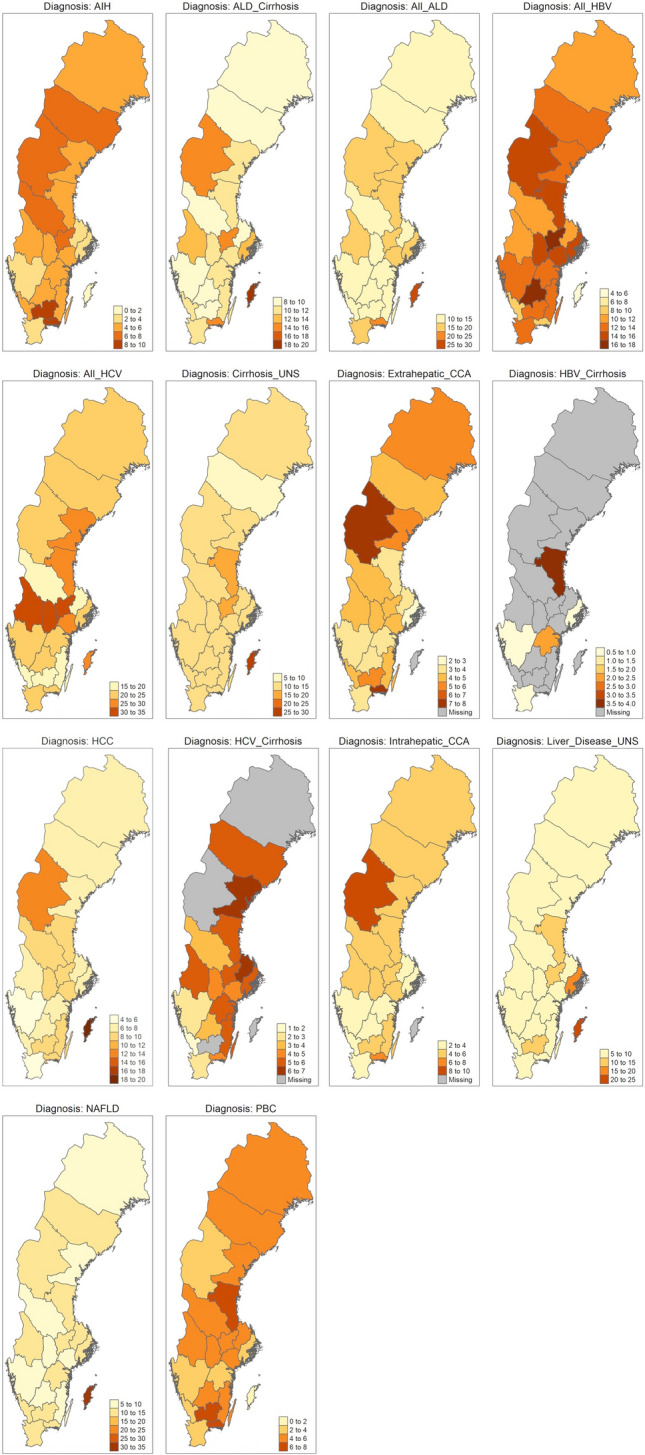
Crude mean annual incidence rate per 100,000 inhabitants for each diagnosis stratified on region, during 2005–2019. Note, different scales are used for the coloring of the heat maps

**Fig. 4:**
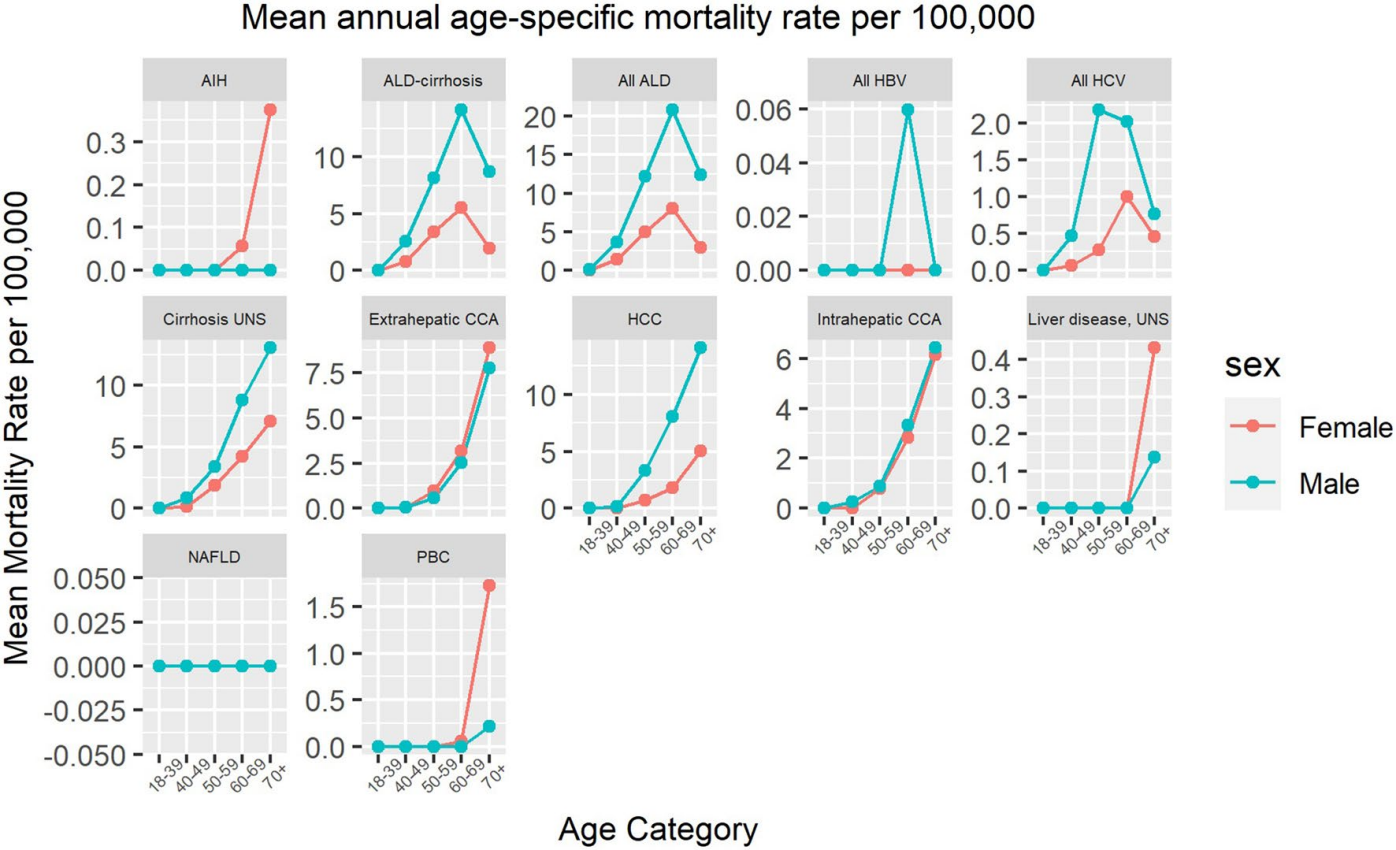
Mean annual age-specific mortality rate per 100,000 inhabitants for each diagnosis, stratified on sex. Note, different scales are used on the y-axes

Age-standardized incidence rates of each liver disease per year, stratified on sex, are presented in Fig. 1. Mean incidence rates during the study period, stratified on categories of age at diagnosis and sex, are presented in Fig. 2. Geographical differences in the crude mean incidence rate during the full study period are presented in Fig. 3. Finally, mean annual mortality rates are presented in Fig. 4 for men and women separately. The main results are summarized below.

Most examined liver diseases increased over time (Fig. 1). The most incident liver diseases in 2019 were ALD (age-standardized incidence rate [ASIR] of 16.4), NAFLD (ASIR of 15.2), and unspecified liver cirrhosis (ASIR of 18.7) (Table 2). Furthermore, the highest annual increases in ASIR were seen for unspecified liver cirrhosis (4.3% annually) and NAFLD (8.0% annually). Most liver diseases were more common in men (Table 2). The disease with the highest mortality rate was alcohol-related liver disease (age-standardized mortality rate [ASMR] of 4.13). Disease-specific data are given below.

#### Alcohol-related liver disease, non-alcoholic fatty liver disease, and unspecified liver disease

Stable trends were observed for ALD (ICD-10: K70), with a mean ASIR of 15.1/100,000 inhabitants and a prevalence in 2019 of 48.7/100,000 inhabitants. We observed a linear increase in the ASIR for ALD-cirrhosis (ICD-10: K70.3) for both sexes during the study period, from 8.9/100,000 (95%CI, 8.3 to 9.5) in 2005, to 13.1/100,000 (95%CI, 12.4 to 13.8) in 2019, with incidence rate ratios (IRRs) between men and women ranging from 2.5 to 3.2 and a mean increase of incidence of 47% (2.6% annually).

Furthermore, we found that the ASIR for unspecified liver disease (ICD-10: K76.9) decreased by 43% (3.6% annually), from 13.3/100,000 (95%CI, 12.6 to 14.1) in 2005, to 7.5/100,000 (95%CI, 7.0 to 8.1) in 2019. A corresponding increase was observed in NAFLD (ICD-10: K76.0), increasing linearly by 217% (8.0% annually), from 4.8/100,000 (95%CI, 4.3 to 5.2) to 15.2/100,000 (95%CI, 14.4 to 15.9), reaching a prevalence of 24.7/100,000 inhabitants in 2019. Moreover, similar trends in age at diagnosis, and in men and women, were seen between these two diagnoses, with both being most frequently diagnosed in individuals aged 60–69, with IRRs for men compared to women ranging from 0.7 to 1.0 for unspecified liver disease and 1.0 to 1.4 for NAFLD. Unspecified liver cirrhosis (ICD-10: K74.6) was most frequently diagnosed in patients older than 70 years of age. The ASIR increased from 10.0/100,000 (95%CI, 9.3 to 10.7) to 18.7/100,000 (95%CI, 17.9 to 19.6), with an IRR for men compared to women of 1.5 at the end of the study period and a mean increase in incidence of 87% (4.3% annually).

#### Viral hepatitis

The incidence rate for viral hepatitis decreased for both HBV (ICD-10: B18.1) and HCV (ICD-10: B18.2). Because HBV-cirrhosis (ICD-10: B18.1E, B18.1G) was uncommon, with a prevalence of 1.5/100,000 inhabitants in 2019, the annual incidence rate is difficult to interpret. However, in HCV-cirrhosis (ICD-10: B18.2E, B18.2G) a clear declining trend was observed during the last six years, from an ASIR for both sexes of 5.1/100,000 (95%CI, 4.7 to 5.6) in 2014 to 2.7/100,000 (95%CI, 2.4 to 3.1) inhabitants, with an IRR for men compared to women ranging from 2.3 to 3.1, reflecting a decrease of 47% (10.1% annually, between 2015 and 2019).

#### Liver malignancies

All included hepatobiliary malignancies showed an increasing trend during the study period. Intrahepatic cholangiocarcinoma (ICD-O-3: C22.1) doubled in ASIR from 1.9/100,000 (95%CI, 1.6 to 2.4) in 2005 to 3.9/100,000 (95%CI, 3.6 to 4.3) inhabitants in 2019, without a sex difference, corresponding to an increase in incidence of 105% (4.9% annually). In individuals diagnosed with HCC (ICD-O-3: C22.0) a strong male predominance was observed, with an IRR of 2.8 compared to women, and an increase in ASIR, from 5.8/100,000 (95%CI, 5.3 to 6.4) to 7.8/100,000 (95%CI, 7.2 to 8.3). During the study period, the increase in incidence was 34% (2.0% annually), with a prevalence of HCC at the end of the study period of 16.0/100,000. In men, an increased ASIR was observed, from 8.3/100,000 (95%CI, 7.4 to 9.2) to 11.4/100,000 (95%CI, 10.5 to 12.4) with an annual increase of 2.1%. Corresponding numbers for women were 3.3/100,000 (95%CI, 2.8 to 3.9) to 4.1/100,000 (95%CI, 3.6 to 4.7) with an annual increase of 1.5%.

#### Autoimmune liver diseases

Between 2005 and 2019, the incidence of PBC (ICD-10: K74.3) was stable with a mean ASIR of 2.3/100,000, and a prevalence of 18.8/100,000 in 2019, being most common in women aged 60–69. A 100% increase of incidence (12.2% annually, between 2005 and 2010) was noted for AIH (ICD-10: K75.4) between 2005 and 2010, increasing from 1.9/100,000 (95%CI, 1.6 to 2.2) to 3.8/100,000 (95%CI, 3.5 to 4.3), before reaching a plateau.

Details on the prevalence as of December 31st, 2019, of the different liver diseases are presented in Table 1. Furthermore, age-standardized incidence rates as well as incidence rate ratios for men compared to women are presented in Table 2.

**Table 1:** Prevalence of each diagnosis on December 31st, 2019, in Sweden. Presented as number of adult persons alive with a diagnosis recorded between 2005 and 2019 in the used registers, per 100,000 individuals and stratified on sex

Diagnosis	Prevalence per 100,000 for both sexes	Prevalence per 100,000 for women	Prevalence per 100,000 for men
All HCV	96.1	61.2	130.6
HCV-cirrhosis	8.1	4.9	11.2
All HBV	87.6	77.5	97.5
HBV-cirrhosis	1.5	0.6	2.5
All ALD	48.7	30.4	66.8
ALD-cirrhosis	40.6	24.5	56.6
Liver cirrhosis, unspecified	44.8	37.9	51.6
NAFLD	24.7	24.2	25.1
AIH	24.3	35.6	13.2
PBC	18.8	32.7	4.9
Liver disease, unspecified	11.3	12.7	9.9
HCC	16.0	8.1	23.8
Intrahepatic CCA	5.7	5.5	5.9
Extrahepatic CCA	4.8	4.4	5.2

*AIH* autoimmune hepatitis, *ALD* alcohol related liver disease, *CCA* cholangiocarcinoma, *HBV* chronic hepatitis B virus, *HCC* hepatocellular carcinoma, *HCV* chronic hepatitis C virus, *NAFLD* non-alcoholic fatty liver disease, *PBC* primary biliary cholangitis

**Table 2:** Age-standardized incidence rate (ASIR) per 100,000 for each diagnosis in 2019, and incidence rate ratio (IRR) between men and women per diagnosis

Diagnosis	Number of incident cases, all	ASIR, all (95%CI)	ASIR, men (95%CI)	ASIR, women (95%CI)	IRR (95%CI) men vs. women
All HCV	1456	14.17 (13.45–14.91)	19.09 (17.92–20.32)	9.18 (8.37–10.05)	2.08 (1.97–2.19)
HCV-cirrhosis	281	2.73 (2.42–3.07)	3.83 (3.32–4.40)	1.62 (1.29–2.01)	2.36 (2.10–2.61)
All HBV	1175	11.43 (10.79–12.10)	13.89 (12.89–14.94)	8.94 (8.14–9.80)	1.55 (1.44–1.67)
HBV-cirrhosis	44	0.43 (0.31–0.58)	0.70 (0.49–0.96)	0.16 (0.07–0.31)	4.45 (3.68–5.21)
All ALD	1687	16.41 (15.64–17.22)	22.50 (21.22–23.83)	10.26 (9.40–11.17)	2.19 (2.09–2.30)
ALD-cirrhosis	1346	13.10 (12.41–13.81)	18.34 (17.19–19.55)	7.79 (7.04–8.59)	2.36 (2.24–2.47)
Liver cirrhosis, unspecified	1923	18.71 (17.88–19.56)	22.52 (21.24–23.85)	14.85 (13.82–15.95)	1.52 (1.43–1.61)
NAFLD	1559	15.17 (14.42–15.94)	15.46 (14.40–16.57)	14.87 (13.84–15.97)	1.04 (0.94–1.14)
AIH	349	3.40 (3.05–3.77)	1.99 (1.63–2.42)	4.81 (4.23–5.46)	0.41 (0.18–0.64)
PBC	288	2.80 (2.49–3.15)	0.97 (0.72–1.28)	4.66 (4.09–5.29)	0.21 (0.00–0.51)
Liver disease, unspecified	771	7.50 (6.98–8.05)	6.77 (6.08–7.52)	8.24 (7.47–9.07)	0.82 (0.68–0.96)
HCC	799	7.77 (7.24–8.33)	11.40 (10.49–12.35)	4.11 (3.57–4.71)	2.77 (2.62–2.93)
Intrahepatic CCA	404	3.93 (3.56–4.33)	3.89 (3.37–4.47)	3.97 (3.45–4.56)	0.98 (0.78–1.17)
Extrahepatic CCA	320	3.11 (2.78–3.47)	3.29(2.81–3.82)	2.94 (2.49–3.44)	1.12 (0.90–1.34)

*AIH* autoimmune hepatitis, *ALD* alcohol related liver disease, *ASIR* age-standardized incidence rate, *CCA* cholangiocarcinoma, *CI* confidence interval, *HBV* chronic hepatitis B virus, *HCC* hepatocellular carcinoma, *HCV* chronic hepatitis C virus, *IRR* incidence rate ratio, *NAFLD* non-alcoholic fatty liver disease, *PBC* primary biliary cholangitis

## Regional differences in incidence rate

We found wide disparities in the crude mean incidence rate of the examined diagnoses across regions (Fig. 3). For instance, the incidences of AIH and PBC were considerably higher in some rural areas as compared to counties with major urban areas (Stockholm, Gothenburg, Malmö). Another example is the island of Gotland, which had the highest rates of both ALD, NAFLD and HCC, but low rates of HBV and cholangiocarcinoma. Data on crude mean annual incidence rate per 100,000 inhabitants for each diagnosis stratified on region, during 2005–2019 is presented in Supplementary Table 2.

### Mortality

#### Alcohol-related liver disease, non-alcoholic fatty liver disease, and unspecified liver disease

The mean annual age-specific mortality rate was similar between deaths attributed to ALD and ALD-cirrhosis with the highest rate observed in men aged 60–69 years. However, the ASMR for both diseases have been stable over the study period with no significant increase observed, neither in total (mean ASMR 4.0/100,000 and 2.7/100,000 inhabitants for ALD and ALD-cirrhosis, respectively) nor stratified on sex (mortality rate ratio [MRR] for ALD and ALD-cirrhosis ranging from 2.3 to 3.8 and from 2.4 to 3.9, respectively, during the study period).

#### Viral hepatitis

The ASMR for HCV was roughly halved during the study period from 0.5/100,000 (95%CI, 0.4 to 0.7) in 2005 to 0.2/100,000 (95%CI, 0.1 to 0.4) in 2019. This trend was mainly attributed to a decreased mortality rate among males, with an ASMR of 0.7/100,000 (95%CI, 0.5 to 1.0) in 2005 to 0.2/100,000 (95%CI, 0.1 to 0.4) in 2019, with an MRR in 2019 of 1.0 (95%CI, 0.2 to 1.8). Only five male individuals had HBV recorded as the main cause of death during the study period.

#### Liver malignancies

Mortality from intrahepatic cholangiocarcinoma (CCA) almost tripled, with an ASMR from 0.7/100,000 (95%CI, 0.5 to 0.9) in 2005 to 1.9/100,000 (95%CI, 1.6 to 2.2) in 2019, without a sex difference. In individuals with HCC coded as the main cause of death, a male predominance was observed, with an MRR ranging from 2.0 to 4.0 during the study period, and with a slight overall increase in ASMR, from 1.5/100,000 (95%CI, 1.3 to 1.8) in 2005 to 2.7/100,000 (95%CI, 2.4 to 3.1) in 2019. This was mainly attributed to an increased ASMR in men (2.3 to 4.2/100,000 men vs. 0.7 to 1.3/100,000 women). Mortality from extrahepatic CCA was stable throughout the study period (mean ASMR 1.6/100,000 inhabitants).

#### Autoimmune liver diseases

During the study period, a mean of 14 (range 9–27) total annual deaths attributable to PBC occurred in Sweden, with an ASMR ranging from 0.1/100,000 to 0.3/100,000 inhabitants, predominantly affecting women aged 70 years or older. Similar trends were observed for AIH (mean 3.2 annual deaths, range 0–11).

Age-standardized mortality rate as well as mortality rate ratios between men and women are presented in Table 3.

**Table 3:** Age-standardized mortality rate (ASMR) per 100,000 for each diagnosis in 2019, and mortality rate ratio (MRR) between men and women per diagnosis

Diagnosis*	Number of deaths, all	ASMR (95%CI)	ASMR, men (95%CI)	ASMR, women (95%CI)	MRR, men vs. women (95%CI)
All HCV	24	0.23 (0.15–0.35)	0.24 (0.12–0.41)	0.24 (0.12–0.41)	0.99 (0.19–1.79)
All ALD	425	4.13 (3.75–4.55)	6.11 (5.46–6.83)	2.13 (1.75–2.57)	2.87 (2.65–3.08)
ALD-cirrhosis	299	2.91 (2.59–3.26)	4.12 (3.59–4.71)	1.68 (1.35–2.08)	2.45 (2.20–2.70)
Liver cirrhosis, unspecified	288	2.80 (2.49–3.16)	3.48 (2.99–4.03)	2.11 (1.73–2.55)	1.65 (1.41–1.89)
PBC	15	0.15 (0.08–0.24)	N/A	0.29 (0.16–0.48)	N/A
Liver disease, unspecified	5	0.05 (0.02–0.11)	N/A	0.10 (0.03–0.23)	N/A
HCC	281	2.73 (2.42–3.07)	4.18 (3.64–4.78)	1.27 (0.98–1.62)	3.29 (3.01–3.56)
Intrahepatic CCA	195	1.90 (1.64–2.18)	2.01 (1.64–2.44)	1.78 (1.43–2.19)	1.13 (0.85–1.41)
Extrahepatic CCA	169	1.64 (1.41–1.91)	1.49 (1.18–1.86)	1.80 (1.45–2.21)	0.83 (0.53–1.13)

^*^There were either a low number (< 5 cases) or no cases of HCV-cirrhosis, HBV, HBV-cirrhosis, NAFLD or AIH listed as the main cause of death in 2019

*AIH* autoimmune hepatitis, *ALD* alcohol related liver disease, *ASMR* age-standardized mortality rate, *CCA* cholangiocarcinoma, *CI* confidence interval, *HBV* chronic hepatitis B virus, *HCC* hepatocellular carcinoma, *HCV* chronic hepatitis C virus, *MRR* mortality rate ratio, *N/A* not applicable, *NAFLD* non-alcoholic fatty liver disease, *PBC* primary biliary cholangitis

## Discussion

In this nationwide observational study, we found important changes in the incidence and mortality rates of register-based diagnoses of chronic liver diseases in Sweden during the last 15 years. An apparent shift in the epidemiology of liver diagnoses has occurred, with an increase in NAFLD and ALD, a two-fold increased incidence rate of AIH, a decrease in viral hepatitis, and an increased burden of liver malignancies. Furthermore, although a decreased incidence rate in unspecified liver disease was observed, the incidence rate of NAFLD increased by 217% (8.0% annually). This could likely be explained by an increased awareness of NAFLD but might also reflect a true increase in incidence. In parallel, a rapid increased in the incidence of unspecified liver cirrhosis was found, corresponding to an increase of 87% (4.3% annually). This is alarming and suggestive of a true increase of cirrhosis related to NAFLD, since the changes in other liver diseases that could lead to the “unspecified liver cirrhosis” diagnosis being made are unlikely to explain this finding (*e.g.,* AIH would be too rare to impact this, and a reduction in the incidence of HCV was seen). However, the mortality rate remained stable for most liver disease etiologies, with ALD and unspecified cirrhosis accounting for most cases of mortality. A decreasing trend in the mortality rate for HCV was observed, while increasing trends were observed in mortality due to intrahepatic CCA and HCC, the latter being mainly attributed to an increase among men.

All data was acquired from national registers and reflect diagnoses acquired from an inpatient or outpatient setting but does not capture diagnoses from primary care. Albeit all included registers have high coverage and validity, some etiologies were likely severely under-coded, especially diseases such as NAFLD, where the prevalence was estimated to 25 cases per 100.000 inhabitants (*i.e.,* ~ 0.03%). Our presented NAFLD prevalence of 0.03% is low in contrast to the meta-analysis by Younossi et al*.* where a prevalence of ~ 25% was estimated [8]. However, in the study by Younossi et al*.,* the prevalence was not based on register-based ICD codes but rather radiological, histological, and blood-based tests, which makes comparison difficult. In a recent study by Alexander et al., data from the European Medical Information Framework from four European countries (*i.e.,* UK, Netherlands, Italy, and Spain) were analyzed based on ICD codes and included more than 18 million European primary health care patients [27]. The estimated prevalence of NAFLD was 0.7%, which in contrast to our results (0.03%) could indicate a higher coverage of NAFLD in primary care than in specialized care-based registers, but in concordance with our findings still reflects low diagnostic awareness compared to an estimated prevalence of 25% [8]. Therefore, the data on incidence and prevalence for NAFLD (and *e.g.,* ALD) should be interpreted with caution. Evidently, diagnoses that are likely to lead to contact with specialized healthcare and unlikely to be misclassified, such as HCC and cirrhosis but also AIH and viral hepatitis, are more likely to be accurately represented in this study.

In this study, we observed a steep decline in the incidence rates of viral hepatitis, which has been shown in previous studies based on the Swedish general population [28]. According to the Public Health Agency of Sweden, the decline in viral hepatitis is likely a consequence of effective direct acting antiviral treatment and needle exchange programs (for HCV) and decreased migration from endemic regions (for HBV) [29]. Also, comparable trends of viral hepatitis related cirrhosis have been observed in many other countries and in tertiary care cohorts from Sweden. For instance, a recent cohort study on all causes of cirrhosis observed a reduction in cirrhosis due to viral hepatitis from 43.4 to 31.0% in 2004 to 2017 while cirrhosis due to NAFLD increased from 5.7 to 14.5% [30]. A similar decrease in viral hepatitis and increase in cirrhosis secondary to NAFLD was reported in the Global Burden of Disease Study in 2017 by Sepanlou et al*.*, whereas compensated and decompensated cirrhosis secondary to NAFLD doubled and tripled, respectively, during the study period (*i.e.,* 1999–2017) [31]. Hence, with an attenuated impact of HBV and HCV, NAFLD and ALD are estimated to be the principal causes of cirrhosis [32], which is supported by our data.

Indeed, we found that unspecified liver cirrhosis and ALD-cirrhosis had a prevalence of 44.8/100,000 and 40.6/100,000 inhabitants, with an ASIR of 18.7/100,000 and 13.1/100,000, respectively. In comparison, HCV- and HBV-cirrhosis had a prevalence of 8.1/100,000 and 1.5/100,000 in 2019. Our incidence rates for unspecified cirrhosis and ALD-cirrhosis portray a higher than previously reported incidence in Sweden [33].

There was an increase both in the incidence and mortality rate for liver malignancies during the study period. This increase could possibly also reflect improved standardized referral pathways for suspected malignancies, increased endoscopic and radiologic diagnostic accuracy, and increased reporting to the SCR. However, with a decreasing incidence of viral hepatitis, this increase could also reflect the increasing incidence of ALD and NAFLD. In a study from a Swedish tertiary centre by Bengtsson et al*.*, NAFLD and ALD constituted approximately 40% of all HCC cases with decreasing trends observed for HCV [34]. Furthermore, at the end of our study period, the ASIR and mortality rate of HCC, intra-, and extrahepatic CCA were in line with a previous study by Akinyemiju et al*.*, that reported incidence and mortality rates of 7/100,000 and 5.5/100,000, respectively, for all liver cancers in Europe [35].

Prevalence, incidence rates, and mortality rates of autoimmune liver diseases are seldom reported due to the rarity of these diseases and the low risk for mortality and liver-related outcomes. During the study period, the mortality rate of PBC was < 0.3/100,000 inhabitants, predominantly seen in elderly women, with similar results for AIH. The prevalence and incidence of PBC was similar as previously reported [36], and stable over time. However, we observed an important increase in the incidence rate for AIH. In a nation-wide population-based cohort study set in Denmark by Grønbæck et al*.*, 1721 AIH patients were identified, with an estimated crude incidence rate of 1.7/100,000, increasing from 1.4/100,000 in 1994 to 2.3/100,000 in 2014, being 2.6 times more common in women [37]. Similar results were observed in a study from New Zealand were the incidence rate increased from 1.4/100,000 to 2.4/100,000 during the study period (*i.e.,* 2008–2016) [38].

Here, we observed a significant increase in the ASIR of AIH between 2005 and 2010, from 1.9/100,000 to 3.8/100,000 inhabitants (before reaching a plateau), with AIH being 2.5 times more common in women. Whether the increased incidence rate of AIH observed throughout different studies is secondary to increased awareness or improved diagnostic accuracy cannot be answered by our study. However, in a recent study by Valgeirsson et al*.*, 71 AIH patients were identified on a national basis during a 10-year period (average population on Iceland during study period ~ 320,000) [39]. The incidence rate was estimated at 2.2/100,000 inhabitants, but a review of medical charts revealed that drug-induced AIH was suspected in 13 out of 71 (18%), with the majority being related to infliximab treatment [39]. Part of the increase in incidence in AIH seen here could possibly be explained by drug-induced AIH but could perhaps also be caused by misclassified drug-induced liver injury secondary to modern immune-activating therapies.

This study has both strengths and limitations. In Sweden, universal health care is tax supported and free of charge, allowing equal and universal access to all levels of health care [40], which limits selection and detection bias due to socioeconomic factors. Also, the study includes all diagnoses of chronic liver diseases between 2005 and 2019 in a single country, based on registers with high coverage and validity [19, 21, 22]. This minimizes risk of missing data from secondary and tertiary level healthcare. However, because primary care is not included in these registers, our data could for some etiologies (for example NAFLD and ALD), primarily capture more severe diagnoses that require more advanced care.

When using register-based data, missing or misclassification of data is always a limitation. Missing data secondary to a chronic liver disease only followed in primary care, or never being diagnosed, can occur. Most patients with a suspected chronic liver disease are recommended to be referred to secondary or tertiary settings for a diagnostic work-up–rendering a diagnosis. However, this is less likely for ALD and NAFLD. Nonetheless, since the awareness of NAFLD in primary care is low, and the ICD code for NAFLD is rarely used, even in the presence of radiologically verified hepatic steatosis, it is unlikely that including data from primary care would have significantly changed our results [41]. Also, inconsistencies between regions in the quality of correct coding of liver diseases might be present. These results highlight the importance in appropriate coding to physicians caring for patients with liver diseases in Sweden and elsewhere. Furthermore, misclassification of ICD codes is a possibility, *e.g.,* AIH with cirrhosis only coded as AIH, or HCV-cirrhosis only coded as HCV. Importantly, since all data was acquired in an aggregated manner, linking data was not possible, hence linking etiologies of unspecified liver diseases or cirrhosis could not be done, and similarly, concomitant comorbidities (*e.g.,* obesity, type 2 diabetes, or cirrhosis) and risk factors (*e.g.,* alcohol consumption) could not be excluded or accounted for. Further, we could not analyze the importance of, or correlation between, hepatic and extrahepatic comorbidities in patients with liver disease due to the use of aggregated data, and such an analysis was therefore out of scope of the research question. Also, when using aggregated data, it is difficult to account for any impact of which *e.g.,* age-period-cohort effects, lead time bias, immigration/emigration, and policy initiatives might have on the results of this study.

We focused on the period between 2005 and 2019 to reduce the risk of inconsistencies in ICD-coding due to changes in the ICD definitions which would lead to the results being difficult to interpret. However, this also affects our estimates of prevalence, since it is possible, but unlikely, that a person could have for instance a diagnosis of AIH only in 2004 and be alive on December 31st, 2019. Such a person would not be captured by our method, but we find such a scenario rare and unlikely to affect our conclusions. Furthermore, in cases with fewer than five individuals per data cell, data was set to missing to protect individual integrity—however, this was only the case when retaining county specific data and did not interfere with any other results. Additionally, we chose to study only a subset of liver diseases. For instance, we did not include primary sclerosing cholangitis, hemochromatosis, Wilson’s disease, or liver transplantation. Finally, all malignancy data was retrieved from the SCR which in general has high coverage and validity [24]. However, in a study by Törner et al*.,* the SCR was found to underestimate HCC and intrahepatic CCA by 35–45% [42]. This could partially be caused by a lack of reporting to the SCR if the liver-related malignancy diagnosis was non-histological (*e.g.,* based on radiological findings). Nonetheless, this strengthens our findings indicating that there has been a true increase in the prevalence and incidence of liver-related malignancies, since, if we would have included non-invasive diagnosis of liver-related malignancies, the numbers could have been even higher.

## Conclusion

In this large register-based study from Sweden we present data on prevalence, incidence, and mortality rates of chronic liver diseases during 2005 to 2019. A shift in the epidemiology of chronic liver disease diagnoses was seen, with incidence rates of NAFLD, ALD-cirrhosis, and unspecified liver cirrhosis increasing while the incidence of viral hepatitis decreased. Furthermore, AIH increased during the study period. Mortality and incidence of liver malignancies is also increasing, likely reflecting the increasing incidence of cirrhosis. We found considerable differences in rates of liver diseases between men and women, but also on a regional level. These results may contribute to allocation of resources by policymakers and clinicians on a regional and national level. Further, although this report depicts shifting rates in the different etiologies of chronic liver diseases, the causative relation of the fluctuating trends cannot be explained with this methodology. The need for adequate surveillance of all chronic liver diseases, including their causes (*e.g.,* obesity, type 2 diabetes, alcohol) and consequences (*e.g.,* liver transplant, cancer, and death), either through robust registers or prospective (collaborative) initiatives, is of importance to identify priorities and health initiatives for both clinicians, researchers, and patients.

## Supplementary Information

Below is the link to the electronic supplementary material.

Supplementary file1 (DOCX 32 kb)

## Data Availability

Upon reasonable request and after required approvals from the Ethics Committee and National Board of Health and Welfare (Socialstyrelsen, Sweden).

## Abbreviations

AIH: Autoimmune hepatitis
ALD: Alcohol-related liver disease
ASIR: Age-standardized incidence rate
ASMR: Age-standardized mortality rate
CCA: Cholangiocarcinoma
CI: Confidence interval
HBV: Chronic hepatitis B virus
HCC: Hepatocellular carcinoma
HCV: Chronic hepatitis C virus
IRR: Incidence rate ratio
NAFLD: Non-alcoholic fatty liver disease
PBC: Primary biliary cholangitis

## Declarations

### Patient and public involvement

No patients were involved in setting the research questions or the outcome measures, nor were they involved in developing plans for the design of the study. No patients were asked to advise on interpretation or writing up of results. There are no plans to disseminate the results of the research to study participants or the relevant patient community.
