# Supplementary material for: Incidence, prevalence and mortality of chronic liver diseases in Sweden between 2005 and 2019
Source: Eur J Epidemiol. 2023 Jul 25;38(9):973–84. doi: 10.1007/s10654-023-01028-x (PMC10501948; doi:10.1007/s10654-023-01028-x)
Supplement: Supplementary file 1 — Supplementary file1 (DOCX 32 kb) [file 10654_2023_1028_MOESM1_ESM.docx]

**Incidence, Prevalence and Mortality of Chronic Liver Diseases in Sweden Between 2005 and 2019**

***Short title:*** *Liver Diseases in Sweden*

*Patrik Nasr, Erik von Seth, Raphaela Mayerhofer, Nelson Ndegwa, Jonas F. Ludvigsson, Hannes Hagström*

# [^patrik.nasr@liu.se^](mailto:patrik.nasr@liu.se)^,^ [^erik.von.seth@ki.se^](mailto:erik.von.seth@ki.se)^,^ [^raphaela.mayerhofer@ki.se^](mailto:raphaela.mayerhofer@ki.se)^,^ [^nelson.ndegwa@gmail.com^](mailto:nelson.ndegwa@gmail.com)^,^ [^jonas.ludvigsson@ki.se^](mailto:jonas.ludvigsson@ki.se)^,^ [^hannes.hagstrom@ki.se^](mailto:hannes.hagstrom@ki.se)

**Supplementary Table 1**: ICD codes used for the study.

| **Diagnosis** | **Patient register (both in- / outpatient)** | **Cancer register** |
| --- | --- | --- |
|  | **ICD-10** | **ICD-O/3.2** |
| Chronic HCV | B18.2 | - |
| HCV-cirrhosis | B18.2E, B18.2G | - |
| Chronic HBV | B18.1 | - |
| HBV-cirrhosis | B18.1E, B18.1G | - |
| ALD | K70.0 | - |
| ALD-cirrhosis | K70.3 | - |
| Liver cirrhosis, unspecified | K74.6 | - |
| NAFLD | K76.0 | - |
| AIH | K75.4 | - |
| PBC | K74.3 | - |
| Liver disease, unspecified | K76.9 | - |
| HCC | - | C22.0 |
| Intrahepatic CCA | - | C22.1 |
| Extrahepatic CCA | - | C24.0 |

**Abbreviations:** AIH, autoimmune hepatitis; ALD, alcohol related liver disease; CCA, cholangiocarcinoma; HBV, chronic hepatitis B virus; HCC, hepatocellular carcinoma; HCV, chronic hepatitis C virus; NAFLD, non-alcoholic fatty liver disease; PBC, primary biliary cholangitis.

**Supplementary Table 2.** Crude mean annual incidence rate per 100,000 inhabitants for each diagnosis stratified by region, during 2005-2019.

|  | All HCV | HCV-cirrhosis | All HBV | HBV-cirrhosis | All ALD | ALD-cirrhosis | Liver cirrhosis, uns | NAFLD | AIH | PBC | Liver disease, uns | HCC | Intra-hepatic CCA | Extra-hepatic CCA |
| --- | --- | --- | --- | --- | --- | --- | --- | --- | --- | --- | --- | --- | --- | --- |
| Stockholm | 22.62 (19.84 - 25.68) | 5.40 (4.09 - 7.01) | 15.59 (13.30 - 18.16) | 0.95 (0.46 - 1.75) | 16.69 (10.58 - 25.04) | 13.63 (11.49-16.05) | 14.28 (12.09 - 16.75) | 11.47 (9.51 - 13.7) | 3.22 (2.23-4.50) | 2.18 (1.38 - 3.27) | 18.3 (15.81 - 21.07) | 7.42 (5.87 - 9.25) | 3.1 (2.13 - 4.36) | 2.07 (1.30 - 3.14) |
| Uppsala | 17.21 (11.57 - 24.64) | 6.18 (3.05 - 11.13) | 10.73 (6.40 - 16.88) | N/A* | 15.01 (10.31 - 21.11) | 9.75 (5.65-15.67) | 12.17 (7.52 - 18.63) | 12.48 (7.79 - 18.97) | 3.62 (1.36-7.77) | 4.09 (1.66 - 8.4) | 5.57 (2.60 - 10.44) | 7.27 (3.83 - 12.54) | 3.72 (1.44 - 7.85) | 3.07 (1.04 – 7.00) |
| Södermanland | 29.66 (21.28 - 40.25) | 4.52 (1.70 - 9.70) | 16.01 (10.05 - 24.22) | N/A* | 11.26 (6.81 - 17.53) | 12.01 (6.94-19.33) | 14.64 (8.97 - 22.56) | 8.35 (4.26 - 14.7) | 5.00 (2.01-10.29) | 4.43 (1.66 - 9.54) | 8.08 (4.05 - 14.41) | 8.4 (4.29 - 14.79) | 5.65 (2.43 - 11.16) | 4.97 (2.00 - 10.23) |
| Östergötland | 22.26 (16.44 - 29.47) | 5.05 (2.54 - 9) | 12.48 (8.24 - 18.14) | 2.35 (0.82 - 5.29) | 14.06 (7.51 - 23.98) | 11.32 (7.30-16.75) | 14.03 (9.50 - 19.96) | 11.56 (7.5 - 17.05) | 4.38 (2.06-8.16) | 3.71 (1.61 - 7.3) | 6.78 (3.77 - 11.22) | 6.45 (3.53 - 10.8) | 3.42 (1.45 - 6.84) | 3.56 (1.52 - 7.08) |
| Jönköping | 21.54 (15.15 - 29.71) | 3.91 (1.55 - 8.15) | 16.25 (10.78 - 23.52) | N/A* | 14.44 (8.42 - 23.09) | 8.38 (4.63-13.97) | 11.73 (7.17 - 18.1) | 7.83 (4.22 - 13.29) | 5.77 (2.74-10.66) | 3.78 (1.46 - 7.99) | 5.31 (2.44 - 10.05) | 6.35 (3.16 - 11.39) | 3.55 (1.33 - 7.63) | 3.86 (1.51 - 8.08) |
| Kronoberg | 15.85 (8.84 - 26.21) | N/A* | 12.84 (6.65 - 22.39) | N/A* | 24.86 (10.12 - 50.83) | 8.61 (3.74-16.90) | 10.95 (5.33 - 19.95) | 14.02 (7.52 - 23.84) | 8.47 (3.67-16.55) | 7.04 (2.74 - 14.8) | 10.57 (5.05 - 19.50) | 7.88 (3.30 - 15.84) | 5.33 (1.73 - 12.43) | 5.5 (1.84 - 12.62) |
| Kalmar | 19.77 (12.58 - 29.57) | 5.12 (1.88 - 11.15) | 12.67 (7.09 - 20.91) | N/A* | 20.77 (11.89 - 33.69) | 10.69 (5.64-18.41) | 11.14 (5.96 - 18.98) | 9.02 (4.45 - 16.27) | 4.73 (1.66-10.56) | 4.64 (1.62 - 10.41) | 9.69 (4.91 - 17.15) | 7.96 (3.72 - 14.89) | 4.96 (1.80 - 10.85) | 4.87 (1.75 - 10.72) |
| Gotland | 29.05 (12.8 - 56.49) | N/A* | 4.19 (0.18 - 20.65) | N/A* | 15.75 (12.81 - 19.17) | 19.10 (6.62-42.94) | 26.15 (10.97 - 52.46) | 31.75 (14.64 - 59.94) | 0 (0-12.77) | 0 (0 - 12.75) | 21.19 (7.88 - 45.74) | 18 (6.05 - 41.2) | N/A* | 7.77 (2.85 - 16.92) |
| Blekinge | 20.56 (11.72 - 33.45) | 4.68 (1.16 - 12.54) | 9.82 (4.13 - 19.67) | N/A* | 14.94 (9.45 - 22.46) | 15.23 (7.83-26.69) | 12.12 (5.65 - 22.73) | 13.54 (6.63 - 24.57) | 7.77 (2.83-16.99) | 6.8 (2.26 - 15.67) | 9.19 (3.71 - 18.88) | 9.68 (4.05 - 19.44) | 6.14 (1.99 - 14.33) | 3.12 (1.90 - 4.83) |
| Skåne | 24.01 (20.34 - 28.16) | 2.27 (1.25 - 3.79) | 13.38 (10.68 - 16.56) | 0.81 (0.26 - 1.89) | 14.19 (11.70 - 17.04) | 10.628.23-13.49) | 11.01 (8.58 - 13.92) | 11.19 (8.73 - 14.13) | 3.35 (2.08-5.11) | 2.75 (1.61 - 4.38) | 8.94 (6.76 - 11.6) | 5.71 (4.00 - 7.91) | 2.34 (1.30 - 3.88) | 4.31 (1.69 - 9.04) |
| Halland | 15.73 (10.08 - 23.4) | 1.69 (0.28 - 5.41) | 8.74 (4.70 - 14.85) | N/A* | 16.1 (10.11 - 24.34) | 11.15 (6.51-17.84) | 11.89 (7.07 - 18.75) | 12.58 (7.60 - 19.61) | 5.13 (2.19-10.17) | 3.47 (1.17 - 7.92) | 6.8 (3.31 - 12.37) | 7.16 (3.57 - 12.81) | 4 (1.5 - 8.58) | 3.67 (2.47 - 5.26) |
| Västra Götaland | 23.18 (19.97 - 26.76) | 2.21 (1.3 - 3.5) | 13.33 (10.92 - 16.1) | 0.59 (0.18 - 1.4) | 14.75 (9.14 - 22.55) | 9.79 (7.75-12.21) | 11.56 (9.33 - 14.17) | 8.18 (6.32 - 10.41) | 3.47 (2.31-5.02) | 2.09 (1.22 - 3.36) | 6.41 (4.78 - 8.41) | 5.99 (4.42 - 7.94) | 3.15 (2.04 - 4.64) | 4.78 (1.86 - 10.04) |
| Värmland | 33.39 (24.44 - 44.54) | 5.75 (2.47 - 11.36) | 11.6 (6.63 - 18.84) | N/A* | 19.46 (12.61 - 28.71) | 12.17 (7.06-19.55) | 14.33 (8.72 - 22.2) | 10.54 (5.83 - 17.52) | 5.52 (2.32-11.06) | 4.59 (1.74 - 9.79) | 7.58 (3.70 - 13.77) | 7.24 (3.46 - 13.32) | 4.55 (1.72 - 9.7) | 4.59 (1.79 - 9.61) |
| Örebro | 30.65 (22.25 - 41.19) | 4.39 (1.66 - 9.36) | 15.5 (9.73 - 23.45) | N/A* | 12.8 (7.57 - 20.27) | 10.48 (5.85-17.30) | 13 (7.77 - 20.41) | 8.40 (4.35 - 14.64) | 5.29 (2.23-10.57) | 5.19 (2.17 - 10.45) | 9.4 (5.06 - 15.95) | 7.69 (3.83 - 13.79) | 4.14 (1.52 - 9.01) | 4.88 (1.83 - 10.49) |
| Västmanland | 30.26 (21.52 - 41.37) | 5.57 (2.30 - 11.3) | 16.64 (10.36 - 25.33) | N/A* | 15.33 (9.54 - 23.35) | 14.14 (8.41-22.28) | 17.21 (10.81 - 26.01) | 11.87 (6.70 - 19.45) | 6.05 (2.60-11.97) | 5.65 (2.33 - 11.44) | 11.46 (6.39 - 18.97) | 9.35 (4.86 - 16.29) | 5.48 (2.24 - 11.18) | 4.15 (1.49 - 9.15) |
| Dalarna | 17.84 (11.54 - 26.36) | 3.62 (1.18 - 8.45) | 11.92 (6.90 - 19.18) | N/A* | 15.48 (9.30 - 24.21) | 9.69 (5.23-16-39) | 13.18 (7.86 - 20.74) | 8.18 (4.15 - 14.47) | 5.66 (2.43-11.18) | 4.21 (1.52 - 9.25) | 7.1 (3.39 - 13.1) | 8.62 (4.46 - 15.05) | 4.56 (1.74 - 9.7) | 3.83 (1.31 - 8.68) |
| Gävleborg | 25.65 (17.95 - 35.54) | 5.51 (2.34 - 10.95) | 15.91 (9.99 - 24.04) | 3.51 (1.14 - 8.19) | 19.38 (10.13 - 33.58) | 11.85 (6.85-19.09) | 16.38 (10.37 - 24.61) | 11.45 (6.55 - 18.6) | 5.30 (2.19-10.73) | 5.85 (2.56 - 11.44) | 12.44 (7.3 - 19.83) | 9.79 (5.31 - 16.52) | 4.65 (1.8 - 9.8) | 5.87 (2.39 - 12.01) |
| Västernorrland | 27.3 (18.83 - 38.29) | 5.97 (2.45 - 12.12) | 13.13 (7.51 - 21.33) | N/A* | 10.74 (5.87 - 18) | 10.96 (5.89-18.61) | 12.69 (7.18 - 20.78) | 7.81 (3.67 - 14.58) | 5.76 (2.32-11.87) | 4.64 (1.64 - 10.33) | 7.63 (3.54 - 14.35) | 8.39 (4.06 - 15.33) | 4.48 (1.54 - 10.13) | 7.9 (2.56 - 18.43) |
| Jämtland | 22.17 (12.16 - 37.11) | N/A* | 14.5 (6.72 - 27.3) | N/A* | 12.4 (7.02 - 20.27) | 14.87 (6.97-27.77) | 14.17 (6.49 - 26.86) | 13.85 (6.28 - 26.44) | 6.82 (1.99-16.88) | 3.88 (0.65 - 12.44) | 10.09 (3.86 - 21.44) | 13.15 (5.82 - 25.5) | 9.5 (3.49 - 20.69) | 5.12 (2.01 - 10.72) |
| Västerbotten | 20.57 (13.54 - 29.94) | 5.41 (2.20 - 11.06) | 12.95 (7.53 - 20.75) | N/A* | 16.69 (10.58 - 25.04) | 7.74 (3.73-14.17) | 9.84 (5.22 - 16.87) | 10.73 (5.87 - 17.99) | 6.46 (2.86-12.53) | 5.54 (2.27 - 11.29) | 7.8 (3.77 - 14.26) | 7.51 (3.58 - 13.87) | 5.44 (2.22 - 11.11) | 5.04 (1.90 - 10.79) |
| Norrbotten | 23.32 (15.64 - 33.46) | N/A* | 10.42 (5.55 - 17.81) | N/A* | 15.01 (10.31 - 21.11) | 8.66 (4.31-15.52) | 10.09 (5.31 - 17.39) | 8.29 (4.03 - 15.08) | 5.93 (2.44-12.05) | 4.26 (1.43 - 9.73) | 7.35 (3.39 - 13.87) | 6.73 (2.98 - 13.04) | 5.56 (2.24 - 11.46) | 2.07 (1.30 - 3.14) |

*There was either a low number (<5 cases) or no cases of the analyzed etiology

**Abbreviations:** AIH, autoimmune hepatitis; ALD, alcohol related liver disease; CCA, cholangiocarcinoma; HBV, chronic hepatitis B virus; HCC, hepatocellular carcinoma; HCV, chronic hepatitis C virus; NAFLD, non-alcoholic fatty liver disease; PBC, primary biliary cholangitis.
